# Efficacy and tolerability of initial low-dose lenvatinib to treat differentiated thyroid cancer

**DOI:** 10.1097/MD.0000000000014774

**Published:** 2019-03-08

**Authors:** Haruhiko Yamazaki, Hiroyuki Iwasaki, Hirotaka Takasaki, Nobuyasu Suganuma, Rika Sakai, Katsuhiko Masudo, Hirotaka Nakayama, Yasushi Rino, Munetaka Masuda

**Affiliations:** a Department of Breast and Endocrine Surgery; b Department of Medical Oncology, Kanagawa Cancer Center; c Department of Breast and Thyroid Surgery, Yokohama City University Medical Center; d Department of Surgery, Yokohama City University School of Medicine, Yokohama City, Kanagawa, Japan.

**Keywords:** efficacy, lenvatinib, thyroid cancer, tolerability

## Abstract

Some patients with differentiated thyroid cancer (DTC) may require an initial low dose (LD) of lenvatinib. However, few studies have investigated the efficacy of LD lenvatinib. We compared the efficacy and tolerability of lenvatinib at an initial LD to those of the standard initial dose of 24 mg in patients with DTC.

In this cross-sectional study, records of patients with DTC treated with lenvatinib were retrospectively reviewed. Patients were divided into 2 groups based on the initial dose of lenvatinib: a full-dose (FD) group that received an initial dose of 24 mg/d and a LD group that received an initial dose of less than 24 mg/d. Categorical variables were compared with the Fisher exact test and continuous variables with Student *t* test. A progression-free survival (PFS) curve was constructed with the Kaplan–Meier method. A probability (*P*) value of < .05 was considered statistically significant.

Thirty-six patients with DTC were treated with lenvatinib (30 in the FD group and 6 in the LD group). The response rates were 43% and 33% in the FD and LD groups, respectively. The median PFS duration was 696 [95% confidence interval (CI): 318–not available (NA)] days in the FD group. The median PFS of the LD group was not reached (95% CI: 124–NA) (*P* = .293). Treatment interruptions were required in 25 (83%) patients in the FD group and 4 (67%) in the LD group (*P* = .573). Dose reductions were required in 28 (93%) patients in the FD group and 4 (67%) in the LD group (*P* = .121). There were no significant differences in the incidences of common adverse events between the 2 groups.

The LD group also required dose reduction and interruption frequently. Since these findings are only the short-term results of a limited number of cases, a large number of cases and long-term observations are needed to determine whether an initial LD is effective for patients with DTC in poor general condition.

## 1 Introduction

About 230,000 new cases of thyroid cancer were diagnosed in 2012 among women and 70,000 among men, with age-standardized (world population) rates of 6.10 and 1.90 per 100,000 persons, respectively.^[1]^ Papillary thyroid carcinoma and follicular thyroid carcinoma are types of differentiated thyroid cancer (DTC), accounting for more than 95% of all thyroid carcinomas.^[2]^ The prognosis of DTC is good, with a disease-specific survival rate greater than 90%.^[3]^ However, the prognosis remains poor for patients with unresectable, advanced, or refractory DTC, with a median 10-year survival rate of 40% to 42%; thus, it is important to develop effective treatment approaches for these patients.^[4]^

Recurrence and distant metastasis of DTCs are treated with radioactive iodine (RAI). The prognosis of patients with I-131 uptake is better than for those without and subsequent treatment options remain limited.^[5]^ So far, no systemic therapy has been found to be effective in controlling DTC and cytotoxic chemotherapy has a limited role in disease management.^[4]^ Although the evidence is very limited, doxorubicin remains the single most effective cytotoxic chemotherapeutic drug to treat DTC.^[6]^ However, sorafenib and lenvatinib, which are multityrosine kinase inhibitors and have high efficacy against RAI-refractory DTCs, have been used in Japan since 2013 and 2015, respectively.^[7,8]^ Although no clinical trial has compared the efficacy of sorafenib with that of lenvatinib yet, the duration of progression-free survival (PFS) and overall response rate were greater with lenvatinib than with sorafenib.^[6]^

Lenvatinib inhibits vascular endothelial growth factor (VEGF) receptors 1, 2, and 3; fibroblast growth factor (FGF) receptors 1to 4; platelet-derived growth factor receptor α (PGDFRα), RET, and KIT signaling networks. In the global phase 3 Study of (E7080) Lenvatinib in Differentiated Cancer of the Thyroid (SELECT), lenvatinib showed a significant antitumor effect, with a median PFS duration of 18.3 and 3.6 months in the lenvatinib and placebo groups, respectively (hazard ratio for progression or death, 0.21; 99% confidence interval (CI), 0.14–0.31; *P* < .001). The response rate, disease control rate, and clinical benefit rate were 64.7%, 87.7%, and 80.1%, respectively. However, some patients discontinued treatment and many required a dose reduction or interruption. The most frequent effects leading to dose discontinuation were asthenia and hypertension, each of which occurred in 1.1% of patients in the lenvatinib group. More patients in the lenvatinib group than in the placebo group required a dose interruption (82.4% vs 18.3%) or reduction (67.8% vs 4.6%). The most common adverse events (AEs) developed during treatment, which led to a dose interruption or reduction among patients receiving lenvatinib, were diarrhea (22.6%), hypertension (19.9%), proteinuria (18.8%), and decreased appetite (18.0%).^[8]^ The results of subgroup analysis showed that Japanese patients were more likely to require a dose reduction (Japanese, 90%; overall, 67.8%).^[9]^

In a phase 1 study, the safety and tolerability of lenvatinib were evaluated in 82 patients with advanced solid tumors refractory to conventional treatments, and the maximum tolerated dose was determined to be 25 mg/d.^[10]^ The results of that study led to the development of a phase II study of lenvatinib to treat advanced RRDTC with an initial dose of 24 mg/d.^[11]^ The standard dose of many oral cancer molecular targeted drugs is equivalent to that for western populations, and the dose is adjusted according to the occurrence of AEs. Resuming lenvatinib at a reduced dose is usually recommended after an interruption, but if the dose at the time of interruption was <14 mg, the same dose should be resumed to maintain the dose intensity.^[12]^ However, some patients start lenvatinib at a low-dose (LD) because of poor performance status or a history of a confounding disease, such as hypertension. No study has yet investigated the efficacy of lenvatinib starting at a LD; thus, the present study aimed to compare the efficacy and tolerability of initial LDs to those of the standard initial dose of 24 mg in patients with DTC.

## 2 Materials and methods

### 2.1 Patients

The protocol of this cross-sectional study was approved by the Institutional Review Board of Kanagawa Cancer Center (Kanagawa, Japan). The records of patients with DTC treated with lenvatinib from May 2015 to September 2018 were reviewed retrospectively. The inclusion criteria were DTC confirmed by histology or cytology, prior total thyroidectomy, age 18 years and older, and use of RAI before treatment with lenvatinib. The exclusion criteria were the coexistence of another thyroid malignancy, and other recurrent or concurrent malignancies.

The following demographic and clinicopathological information was collected for analysis: age, sex, Eastern Cooperative Oncology Group performance status, height, body weight, histological type of thyroid cancer, previous tyrosine kinase inhibitor treatment, initial dose of lenvatinib, reported treatment interruptions or dose reductions, distant metastasis sites, clinical response to lenvatinib, PFS duration, and AEs. Patients were divided into 2 groups based on the initial dose of lenvatinib: a full-dose (FD) group that received an initial dose of 24 mg/d and a LD group that received an initial dose of less than 24 mg/d. The reasons for choosing an LD of lenvatinib were also collected.

### 2.2 Objectives

The objectives of this study were to compare the clinical response, PFS duration, rates of lenvatinib interruption and dose reduction, and incidence of AEs between the 2 groups.

### 2.3 Definitions

PFS was calculated as the time from the start of lenvatinib to disease progression or the date of death from any cause. The clinical response to lenvatinib was evaluated by computed tomography using the Response Evaluation Criteria in Solid Tumors, version 1.1.^[13]^

AEs were evaluated using the Common Terminology Criteria for Adverse Events, version 4.0.

### 2.4 Statistical analysis

All statistical analyses were performed with EZR (Saitama Medical Center, Jichi Medical University, Saitama, Japan), which is a graphical user interface for R (The R Foundation for Statistical Computing, Vienna, Austria). More precisely, it is a modified version of R commander designed to add statistical functions used frequently in biostatistics. Comparisons were made between the 2 groups to identify any significant imbalances in patient characteristics. Categorical variables were compared with the Fisher exact test and continuous variables with Student *t* test. A PFS curve was constructed with the Kaplan–Meier method. A probability (*P*) value of <.05 was considered statistically significant. For the survival analysis, the final examination date was confirmed with the medical record. There were no patients who were lost to follow-up.

## 3 Results

### 3.1 Study population

Thirty-six patients with DTC were treated with lenvatinib: 30 patients in the FD group and 6 in the LD group. The baseline characteristics of the 2 groups are shown in Table 1. All baseline characteristics were comparable between the 2 groups except for height, which was significantly lower in the LD group (*P* = .03). The initial dose of lenvatinib in the LD group was 20 mg/d for 3 patients, 14 mg/d for 1, and 10 mg/d for 2. The setting of the initial dose was based on the physician's choice, and there was no uniformity. The reasons for starting with an LD were hypertension in 3 cases, advanced age in 2, and a concern of fistula formation in 1. The median follow-up time was 510 days (range, 40–1210) in the FD group and 366 days (range, 124–740) in the LD group.

**Table 1:**
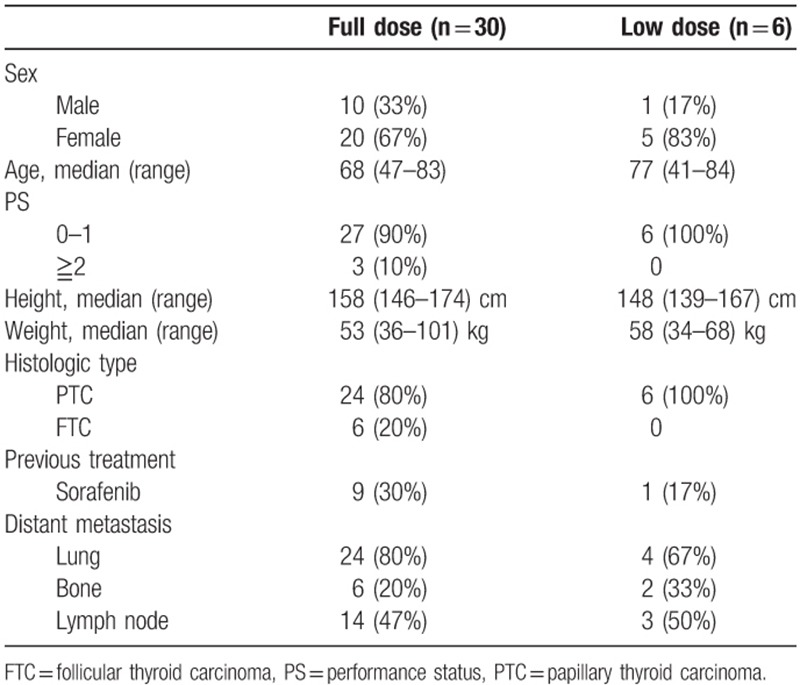
Patient characteristics.

### 3.2 Efficacy

**Figure 1:**
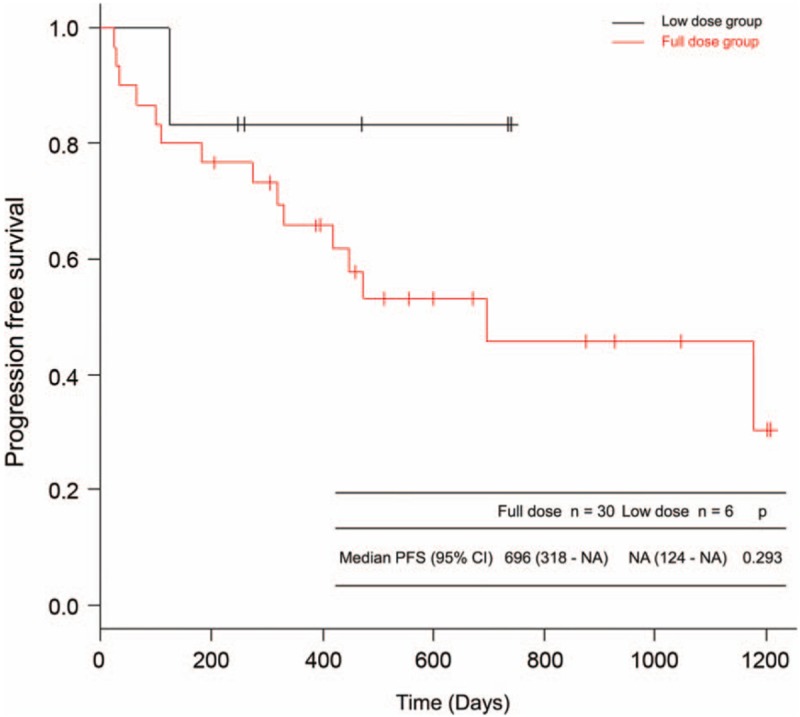
PFS of the FD and LD groups. The median PFS duration was 696 (95% CI: 318–NA) days in the FD group. The median PFS of the LD group was not reached (95% CI: 124–NA) (*P* = .293). CI = confidence interval, FD = full dose, LD = low dose, NA = not available, PFS = progression-free survival.

The response rates were 43% and 33% in the FD and LD groups, respectively. The median PFS duration was 696 [95% CI: 318–not available (NA)] days in the FD group. The median PFS of the LD group was not reached (95% CI: 124–NA) (*P* = .293) (Fig. 1).

### 3.3 Tolerability profile

A summary of the rates of lenvatinib dose reduction and interruption, and the incidence of AEs are shown in Table 2. Dose reduction was required in 28 (93%) patients in the FD group and 4 (67%; 3 patients from 20 mg and 1 from 14 mg) in the LD group (*P* = .121). Treatment interruption was required in 25 (83%) patients in the FD group and 4 (67%; 2 patients from 20 mg, 1 from 14 mg, and 1 from 10 mg) in the LD group (*P* = .573). There were no significant differences in the incidences of hypertension, proteinuria, fatigue, anorexia, diarrhea, and palmar–plantar erythrodysesthesia syndrome, which are common AEs of lenvatinib, between the 2 groups. There were no serious AEs resulting in hospitalization or death.

**Table 2:**
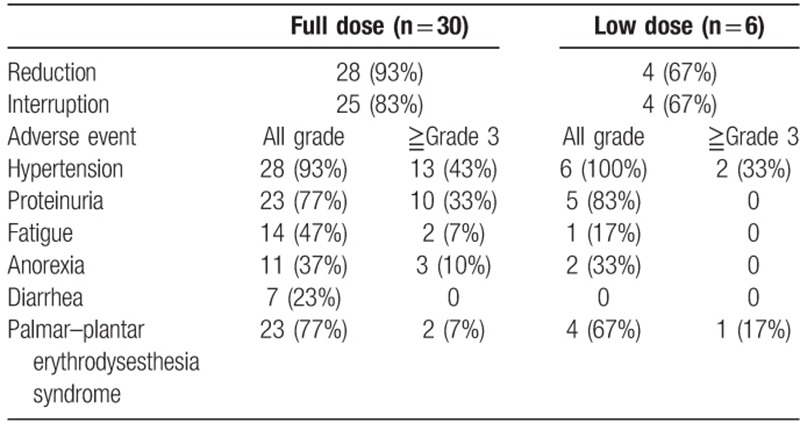
Tolerability and adverse events.

## 4 Discussion

In this study, the efficacy and tolerability of an initial LD were compared with those of the standard initial dose of 24 mg/d to treat DTC. The data showed that 2 patients who were treated by initial 20 mg dose had a partial response, suggesting that an initial LD might be effective. However, the rates of lenvatinib dose reduction and interruption, and the incidence of AEs were high in the LD group.

Comparisons of different initial doses of sorafenib, as a molecular targeted therapy for thyroid cancer, have been reported. There were no significant differences in the efficacy and tolerability among 51 cases treated with an initial FD and 24 cases starting with an LD. The median durations of overall survival were 56 and 30 months in the FD and LD groups, respectively. Although there was no statistically significant difference, overall survival tended to be poorer in the LD group. As a possible explanation, patients in poorer general condition were included in the LD group.^[14]^

Lenvatinib has significant antitumor effects and is used to treat RRDTC, unresectable anaplastic thyroid cancer, and unresectable medullary thyroid cancer in Japan. In this study, the records of 36 patients with RRDTC treated with lenvatinib were reviewed retrospectively. In clinical practice, the initial dose of lenvatinib is determined by the patient's characteristics. Suyama et al^[15]^ reported that of 20 patients with DTC treated with lenvatinib, 5 started lenvatinib at an LD due to vessel invasion, trachea invasion, and advanced age. Berdelou et al^[16]^ reported that of 75 patients, 54 started lenvatinib at a FD and 21 at an LD. The reasons of starting at an LD were comorbidity in 1 case, advanced age in 2, poor performance status in nine, and unknown in nine. The response rate was nearly equal between the LD and FD groups (28% vs 31%, respectively).^[16]^ In this study, 6 patients started lenvatinib at an LD, but the efficacy was not significantly different between the 2 groups. The reasons for starting at an LD were similar to past reports.

In subgroup analysis of the SELECT, Japanese patients were more likely to require a dose reduction (Japanese, 90%; overall, 67.8%).^[9]^ In the present study, dose reduction and interruption were also required in many cases, but there were no significant differences in the rates of reduction and interruption between the 2 groups. With other molecular targeted agents, no correlation was found between the dose intensity and efficacy in terms of PFS.^[17,18]^ We think that there are some RRDTC patients who have efficacy of lenvatinib at a low dose.

According to the results of a phase II study, the optimal initial dosage of lenvatinib for patients with hepatocellular carcinoma is 12 mg at a body weight of 60 kg or more and 8 mg at a body weight less than 60 kg.^[19]^ In a phase III trial, lenvatinib was not inferior to sorafenib in treating hepatocellular carcinoma.^[20]^ Population pharmacokinetic analysis reported that body weight is not considered clinically relevant; thus, a dose adjustment of lenvatinib is not warranted.^[21]^ However, higher toxicity was observed in older patients in the SELECT.^[22]^ Therefore, it is appropriate to change the initial dose according to the patient's age.

There were some limitations to this study. First, this study was not prospective or randomized. Therefore, the patient characteristics differed between the 2 groups. Second, because the initial dose of the LD group was different, drug efficacy could not be evaluated uniformly. Third, the number of cases in the 2 groups was small and the observation period was short. We think that the short observation period interfered in measuring the true PFS time of low dose group.

## 5 Conclusion

The LD group also required dose reduction and interruption frequently. Since these findings are only the short-term results of a limited number of cases, a large number of cases and long-term observations are needed to determine whether an initial LD is effective for patients with RRDTC in poor general condition.

## Acknowledgments

The authors thank Enago (https://www.enago.jp/) for editing this manuscript.

## Author contributions

**Data curation:** Hiroyuki Iwasaki, Hirotaka Takasaki, Nobuyasu Suganuma, Rika Sakai.

**Supervision:** Hirotaka Nakayama, Katsuhiko Masudo, Yasushi Rino, Munetaka Masuda.

**Writing – original draft:** Haruhiko Yamazaki.

**Writing – review & editing:** Haruhiko Yamazaki.

Haruhiko Yamazaki orcid: 0000-0002-6640-3529.

## References

[1] La Vecchia C Malvezzi M Bosetti C Thyroid cancer mortality and incidence: a global overview. Int J Cancer 2015;136:2187–95. 25284703 10.1002/ijc.29251

[2] Aschebrook-Kilfoy B Ward MH Sabra MM Thyroid cancer incidence patterns in the United States by histologic type, 1992-2006. Thyroid 2011;21:125–34. 21186939 10.1089/thy.2010.0021 PMC3025182

[3] Matsuzu K Sugino K Masudo K Thyroid lobectomy for papillary thyroid cancer: long-term follow-up study of 1,088 cases. World J Surg 2014;38:68–79. 24081532 10.1007/s00268-013-2224-1

[4] Cabanillas ME Habra MA Lenvatinib: role in thyroid cancer and other solid tumors. Cancer Treat Rev 2016;42:47–55. 26678514 10.1016/j.ctrv.2015.11.003

[5] Durante C Haddy N Baudin E Long-term outcome of 444 patients with distant metastases from papillary and follicular thyroid carcinoma: benefits and limits of radioiodine therapy. J Clin Endocrinol Metab 2006;91:2892–9. 16684830 10.1210/jc.2005-2838

[6] Lorusso L Pieruzzi L Biagini A Lenvatinib and other tyrosine kinase inhibitors for the treatment of radioiodine refractory, advanced, and progressive thyroid cancer. Onco Targets Ther 2016;9:6467–77. 27799794 10.2147/OTT.S84625 PMC5079697

[7] Brose MS Nutting CM Jarzab B Sorafenib in radioactive iodine-refractory, locally advanced or metastatic differentiated thyroid cancer: a randomised, double-blind, phase 3 trial. Lancet 2014;384:319–28. 24768112 10.1016/S0140-6736(14)60421-9 PMC4366116

[8] Schlumberger M Tahara M Wirth LJ Lenvatinib versus placebo in radioiodine-refractory thyroid cancer. N Engl J Med 2015;372:621–30. 25671254 10.1056/NEJMoa1406470

[9] Kiyota N Schlumberger M Muro K Subgroup analysis of Japanese patients in a phase 3 study of lenvatinib in radioiodine-refractory differentiated thyroid cancer. Cancer Sci 2015;106:1714–21. 26426092 10.1111/cas.12826 PMC4714672

[10] Yamada K Yamamoto N Yamada Y Phase I dose-escalation study and biomarker analysis of e7080 in patients with advanced solid tumors. Clin Cancer Res 2011;17:2528–37. 21372218 10.1158/1078-0432.CCR-10-2638

[11] Cabanillas ME Schlumberger M Jarzab B A phase 2 trial of lenvatinib (E7080) in advanced, progressive, radioiodine-refractory, differentiated thyroid cancer: a clinical outcomes and biomarker assessment. Cancer 2015;121:2749–56. 25913680 10.1002/cncr.29395 PMC4803478

[12] Takahashi S Kiyota N Tahara M Optimal use of lenvatinib in the treatment of advanced thyroid cancer. Cancers Head Neck 2017;2:7. 10.1186/s41199-017-0026-0 PMC6460646 31093354

[13] Therasse P Arbuck SG Eisenhauer EA New guidelines to evaluate the response to treatment in solid tumors. European Organization for Research and Treatment of Cancer, National Cancer Institute of the United States, National Cancer Institute of Canada. J Natl Cancer Inst 2000;92:205–16. 10655437 10.1093/jnci/92.3.205

[14] Dadu R Waguespack SG Sherman SI Efficacy and tolerability of different starting doses of sorafenib in patients with differentiated thyroid cancer. Oncologist 2014;19:477–82. 24733667 10.1634/theoncologist.2013-0409 PMC4012968

[15] Suyama K Tomiguchi M Takeshita T Factors involved in early lenvatinib dose reduction: a retrospective analysis. Med Oncol 2018;35:19. 29387983 10.1007/s12032-018-1088-5

[16] Berdelou A Borget I Godbert Y Lenvatinib for the treatment of radioiodine-refractory thyroid cancer in real-life practice. Thyroid 2018;28:72–8. 10.1089/thy.2017.0205 29048237

[17] Ciccarese M Fabi A Moscetti L Dose intensity and efficacy of the combination of everolimus and exemestane (EVE/EXE) in a real-world population of hormone receptor-positive (ER+/PgR+), HER2-negative advanced breast cancer (ABC) patients: a multicenter italian experience. Breast Cancer Res Treat 2017;163:587–94. 28353061 10.1007/s10549-017-4213-9

[18] Masuda N Nishimura R Takahashi M Palbociclib in combination with letrozole as first-line treatment for advanced breast cancer: a Japanese phase II study. Cancer Sci 2018;109:803–13. 29345736 10.1111/cas.13507 PMC5834809

[19] Tamai T Hayato S Hojo S Dose finding of lenvatinib in subjects with advanced hepatocellular carcinoma based on population pharmacokinetic and exposure-response analyses. J Clin Pharmacol 2017;57:1138–47. 28561918 10.1002/jcph.917 PMC5575539

[20] Kudo M Finn RS Qin S Lenvatinib versus sorafenib in first-line treatment of patients with unresectable hepatocellular carcinoma: a randomised phase 3 non-inferiority trial. Lancet 2018;391:1163–73. 29433850 10.1016/S0140-6736(18)30207-1

[21] Gupta A Jarzab B Capdevila J Population pharmacokinetic analysis of lenvatinib in healthy subjects and patients with cancer. Br J Clin Pharmacol 2016;81:1124–33. 26879594 10.1111/bcp.12907 PMC4876185

[22] Brose MS Worden FP Newbold KL Effect of age on the efficacy and safety of lenvatinib in radioiodine-refractory differentiated thyroid cancer in the phase III SELECT Trial. J Clin Oncol 2017;35:2692–9. 28613956 10.1200/JCO.2016.71.6472

